# Ixodid ticks in red foxes (*Vulpes vulpes*) from Romania

**DOI:** 10.1186/1756-3305-7-S1-P1

**Published:** 2014-04-01

**Authors:** MO Dumitrache, G D'Amico, IA Matei, A Ionică, CM Gherman, S Sikó Barabási, DT Ionescu, M Oltean, A Balea, IC Ilea, AD Sándor, AD Mihalca

**Affiliations:** 1 Department of Parasitology and Parasitic Diseases, Faculty of Veterinary Medicine, University of Agricultural Sciences and Veterinary Medicine, Cluj-Napoca, România; 2 Department of Environmental Science, Faculty of Environmental Science, Babeș-Bolyai University, Sfântu Gheorghe, România; 3 Department of Game and Wildlife, Faculty of Silviculture and Forestry Engineering, Transilvania University, Brașov, România; 4 Department of Congress Publication Coordinator, XPE Pharma&Science, Cluj-Napoca, România

## 

Among wildlife, the red foxes (*Vulpes vulpes*) are one of the most adapted wild species to anthropic ecosystems. They are well recognized as important reservoirs for a large number of zoonotic agents in Europe, including ticks and tick-borne pathogens. Currently, there are few available data on the importance of red foxes in the ecoepidemiology of vector-borne diseases. Therefore, the aim of this study was to screen the dynamics of tick infestation in 357 red foxes from 12 Romanian counties. Tick identification was performed using the morphological keys. The overall prevalence of tick infestation was 43.7%. The 5753 collected ticks belonged to five species: *Ixodes hexagonus *(on 113 out of 156 foxes; prevalence 72.44%), *I. ricinus *(28.84%), *I. crenulatus *(7.7%), *Dermacentor marginatus *(7.05%) and *Haemaphysalis punctata *(0.64%). Coinfestation occurred in 24 foxes (22 with 2 tick species; 2 with 3 tick species) with the following associations: *I. ricinus + I. hexagonus *(n = 10), *I. hexagonus + D. marginatus *(n = 5), *I. ricinus + I. crenulatus *(n = 4), *I. ricinus + D. marginatus *(n = 2), *I. hexagonus + I. crenulatus *(n = 1), *D. marginatus + I. hexagonus + I. ricinus *(n = 1), and *H. punctata + I. hexagonus + I. ricinus *(n = 1). This study indicates that foxes are hosts to a relevant number of tick species with recognized vectorial role. The provided information can facilitate the understanding of the ecology of ticks and can be the basis for studies on the epidemiology of tick-borne diseases. The high prevalence of tick infestation in red foxes, coupled with the increasing presence of this species in synanthropic environments and a more nature-oriented lifestyle of people, can pose a significant threat to human health, through the emergence of tick-borne diseases. Moreover, foxes represent a good model of sentinel species.

This research was performed as part of project IDEI PCE 236/2011.

